# Antenatal Diagnosis and Management of Congenital Pulmonary Airway Malformation: A Case Report

**DOI:** 10.7759/cureus.102859

**Published:** 2026-02-02

**Authors:** Sreedevi Sathian, Dhiyanath Pattanath, Jisha Chullikkatil

**Affiliations:** 1 Department of Radiology, Koya's Hospital, Kozhikode, IND; 2 Department of Radiodiagnosis, Government Medical College Calicut, Kozhikode, IND; 3 Department of Obstetrics and Gynaecology, Koya's Hospital, Kozhikode, IND

**Keywords:** antenatal corticosteroid, congenital pulmonary airway malformation, cpam volume ratio, non-immune hydrops fetalis, polyhydramnios

## Abstract

Congenital pulmonary airway malformation (CPAM) is a rare developmental lung anomaly that can be identified on prenatal ultrasonography. We report a case of antenatally diagnosed CPAM with a progressively increasing CPAM volume ratio (CVR) on serial foetal imaging. Antenatal corticosteroids were administered, and a male neonate was delivered by elective lower-segment caesarean section at 33 weeks of gestation. Postnatal imaging confirmed CPAM involving the right middle lobe. The infant underwent right middle lobectomy on postnatal day nine with an uneventful postoperative course. This case highlights the importance of serial CVR monitoring, antenatal planning, and timely surgical management in achieving favourable outcomes in high-risk CPAM.

## Introduction

Congenital pulmonary airway malformation (CPAM) is an uncommon congenital lung anomaly resulting from abnormal bronchial development, characterised by disorganised proliferation of terminal bronchioles and replacement of normal lung parenchyma with cystic or solid dysplastic tissue [1].

The condition was initially described by Ch’in and Tang in 1949 as congenital cystic adenomatoid malformation [2]. Stocker et al. later introduced the term CPAM and proposed a revised classification comprising five subtypes based on airway level of origin and histopathological features [3]. CPAM affects male and female foetuses equally and is usually unilateral, with a predilection for lower-lobe involvement [3]. Antenatal associations include mediastinal shift, polyhydramnios, pleural effusion, and, in severe cases, non-immune hydrops fetalis [4].

This is a case of antenatally diagnosed CPAM with severe polyhydramnios that was managed successfully with favourable foetal and maternal outcomes through multidisciplinary care despite late presentation and high-risk features.

## Case presentation

A pregnant woman with a history of two previous lower-segment caesarean sections (LSCS) was referred at 33 weeks of gestation, after a routine obstetric ultrasound detected a large right-sided foetal multicystic lung mass. Targeted foetal ultrasonography demonstrated imaging features consistent with CPAM, associated with a significant mediastinal shift.

**Figure 1:**
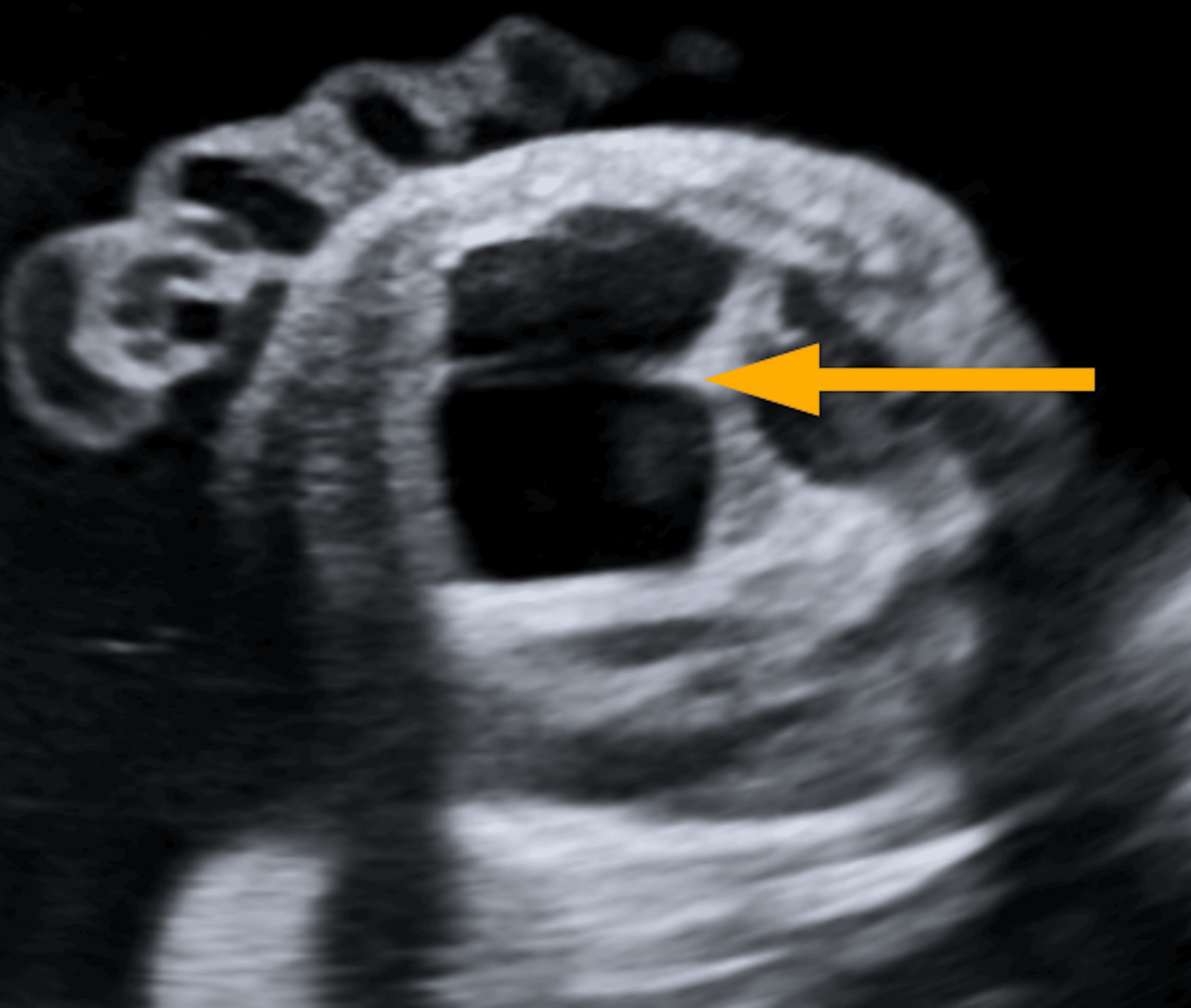
Prenatal ultrasound image demonstrating macrocystic congenital pulmonary airway malformation (arrow), characterised by multiple large cystic spaces within the foetal lung.

**Figure 2:**
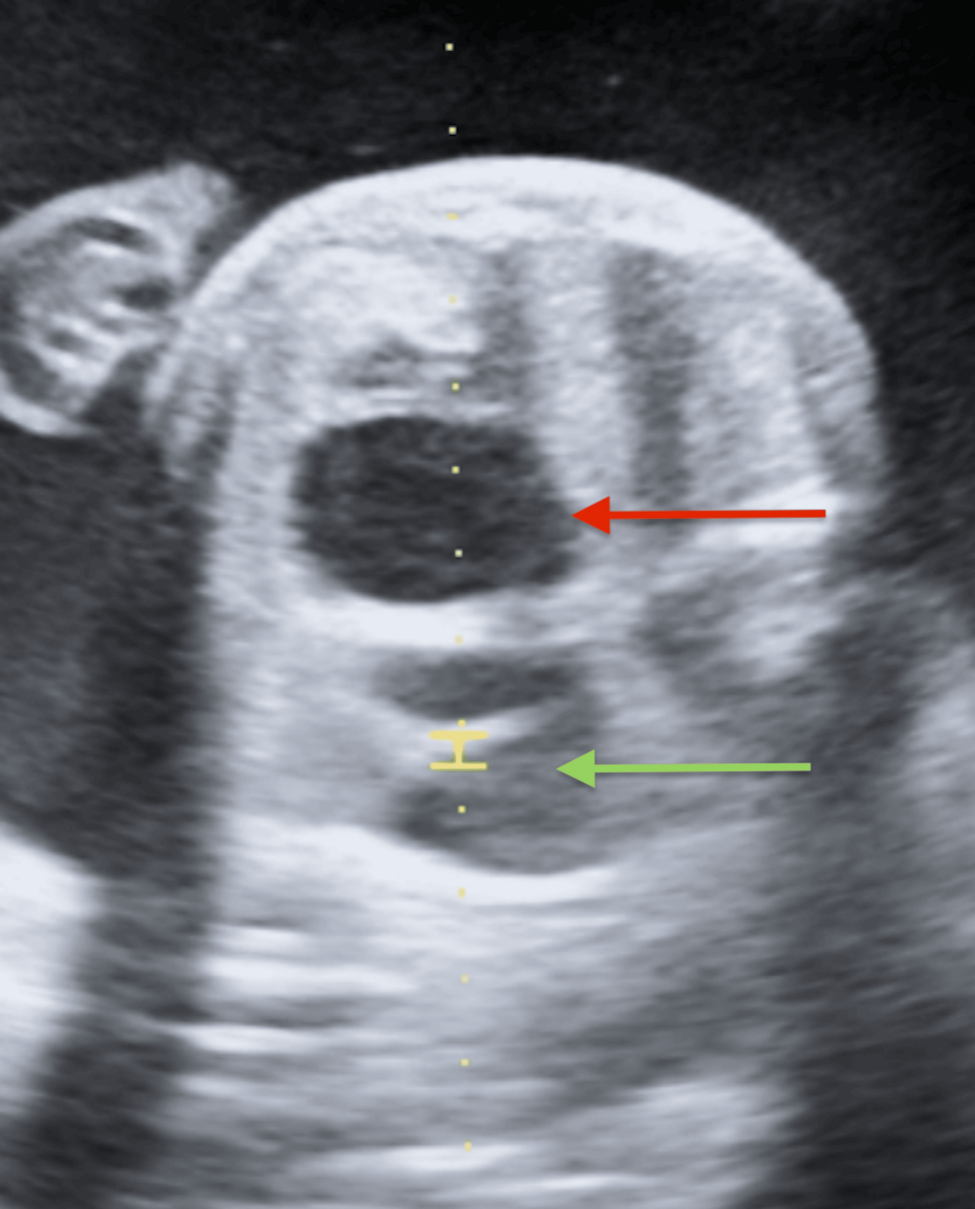
Prenatal ultrasound image demonstrating macrocystic CPAM (red arrow) of the foetal lung with large cystic components causing contralateral mediastinal shift (green arrow). CPAM: congenital pulmonary airway malformation.

**Figure 3:**
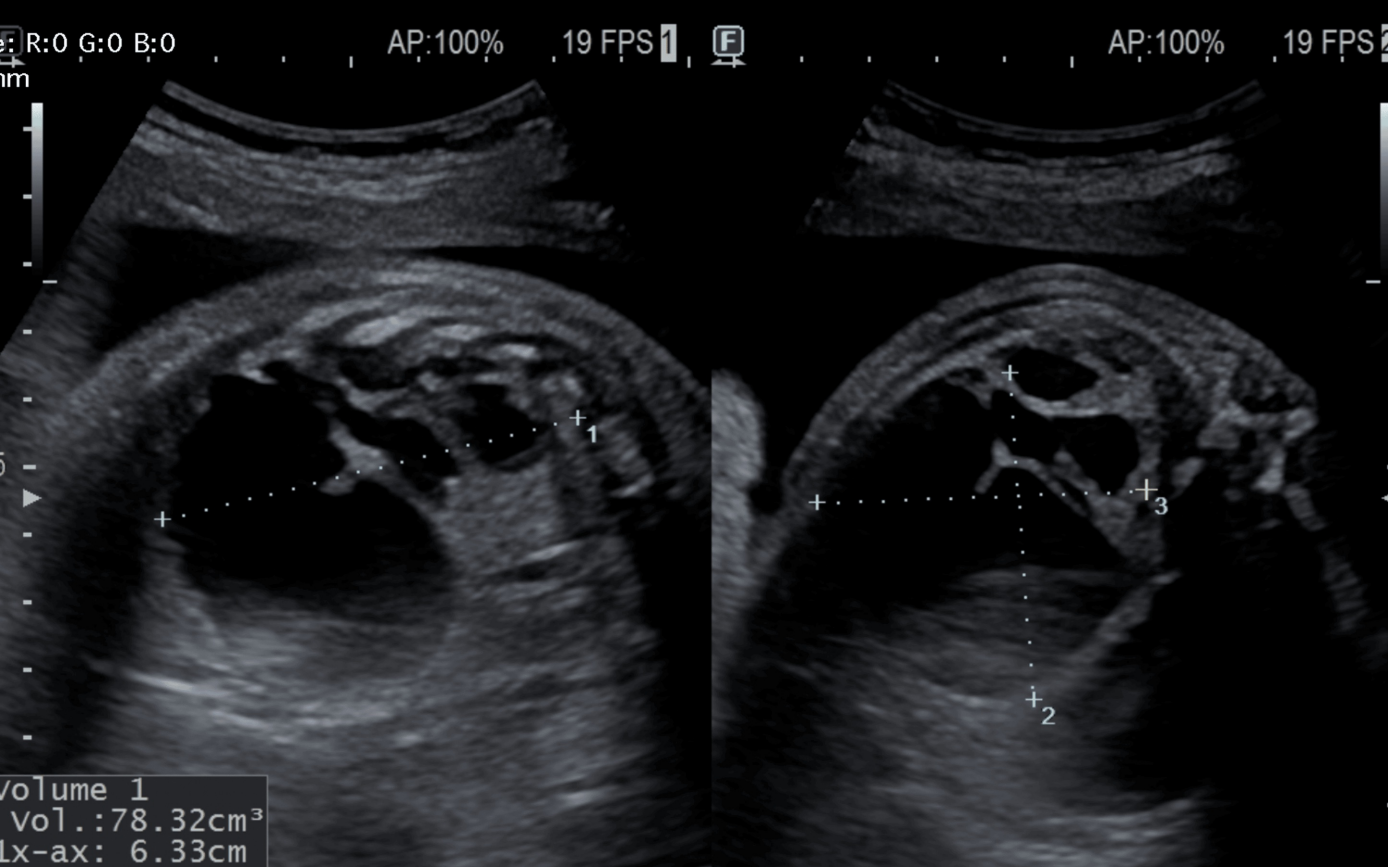
Prenatal ultrasound image demonstrating CPAM volume calculation, obtained by measuring the maximum length, width, and height of the lesion and applying the ellipsoid formula. CPAM: congenital pulmonary airway malformation.

Gross polyhydramnios was noted, with an amniotic fluid index (AFI) of 37 cm. The volume of the multicystic lung lesion was calculated to be 78 cc, with a CPAM volume ratio (CVR) of 2.5, placing the foetus in a high-risk category for the development of hydrops fetalis. The polyhydramnios was attributed to oesophageal compression from the mass effect, mediastinal shift, impaired foetal swallowing, and compromised venous return (Figures 1-3). Image quality was reduced due to polyhydramnios. Serial antenatal ultrasound examinations were performed, and the CVR was calculated, which rose to 2.8.

Despite the late presentation at 33 weeks, the patient was administered antenatal corticosteroid (betamethasone) in view of the high CVR, and the high-risk obstetric team was alerted for close surveillance and multidisciplinary management. A male baby was born out of elective LSCS at 33 weeks in view of the previous two LSCS and polyhydramnios. The APGAR score was 7 at one and five minutes. The baby’s heart rate was 156 beats/min, respiratory rate was 66 breaths/min, and decreased air entry was noted over the right hemithorax. The birth weight was 2.4 kg. The baby was shifted to the neonatal intensive care unit for monitoring and respiratory support via bubble continuous positive airway pressure (BCPAP).

**Figure 4:**
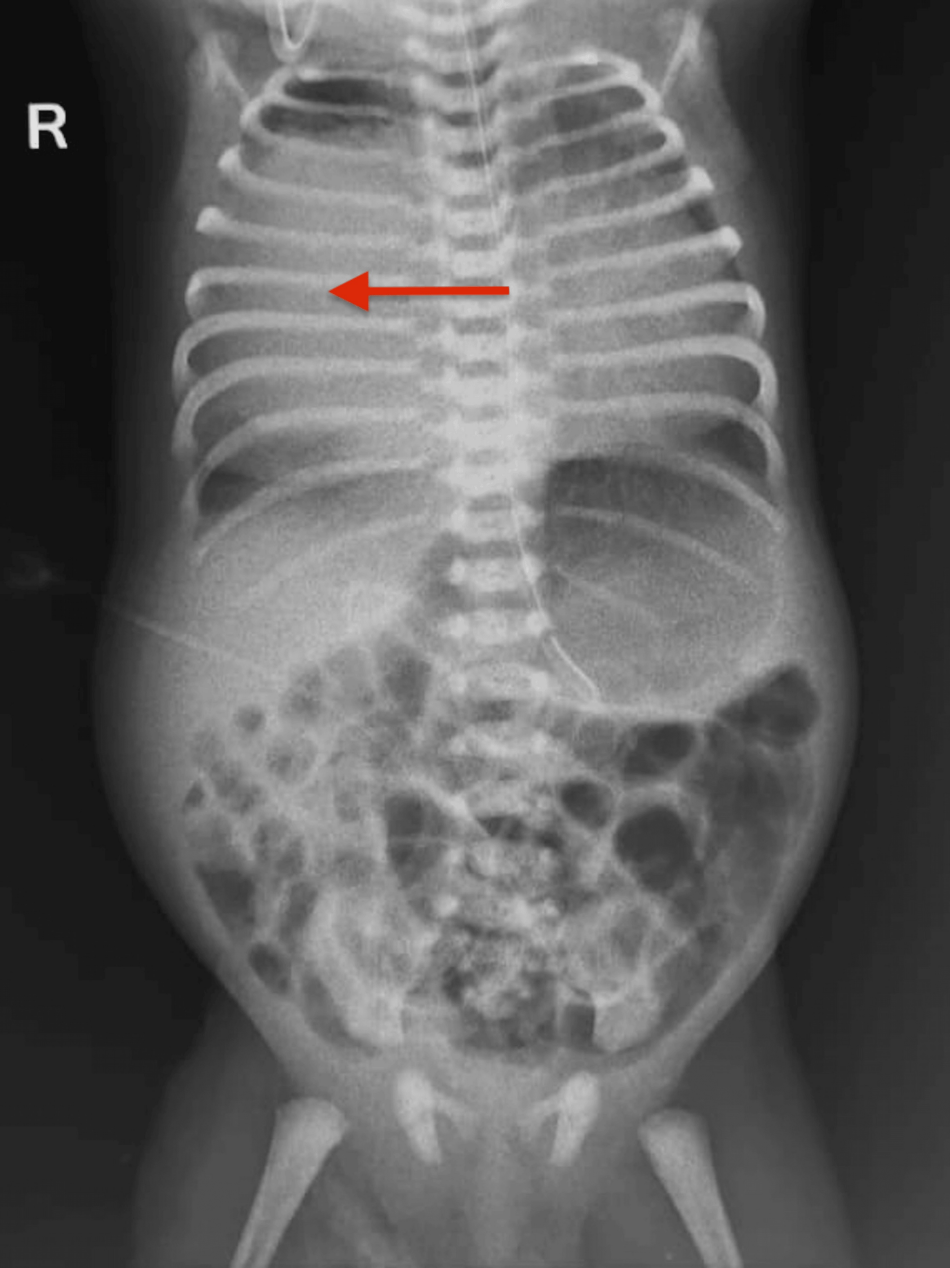
Chest X-ray showing a well-defined, homogeneous opacity (arrow) predominantly involving the middle zone of the right lung, causing a tracheomediastinal shift to the left.

**Figure 5:**
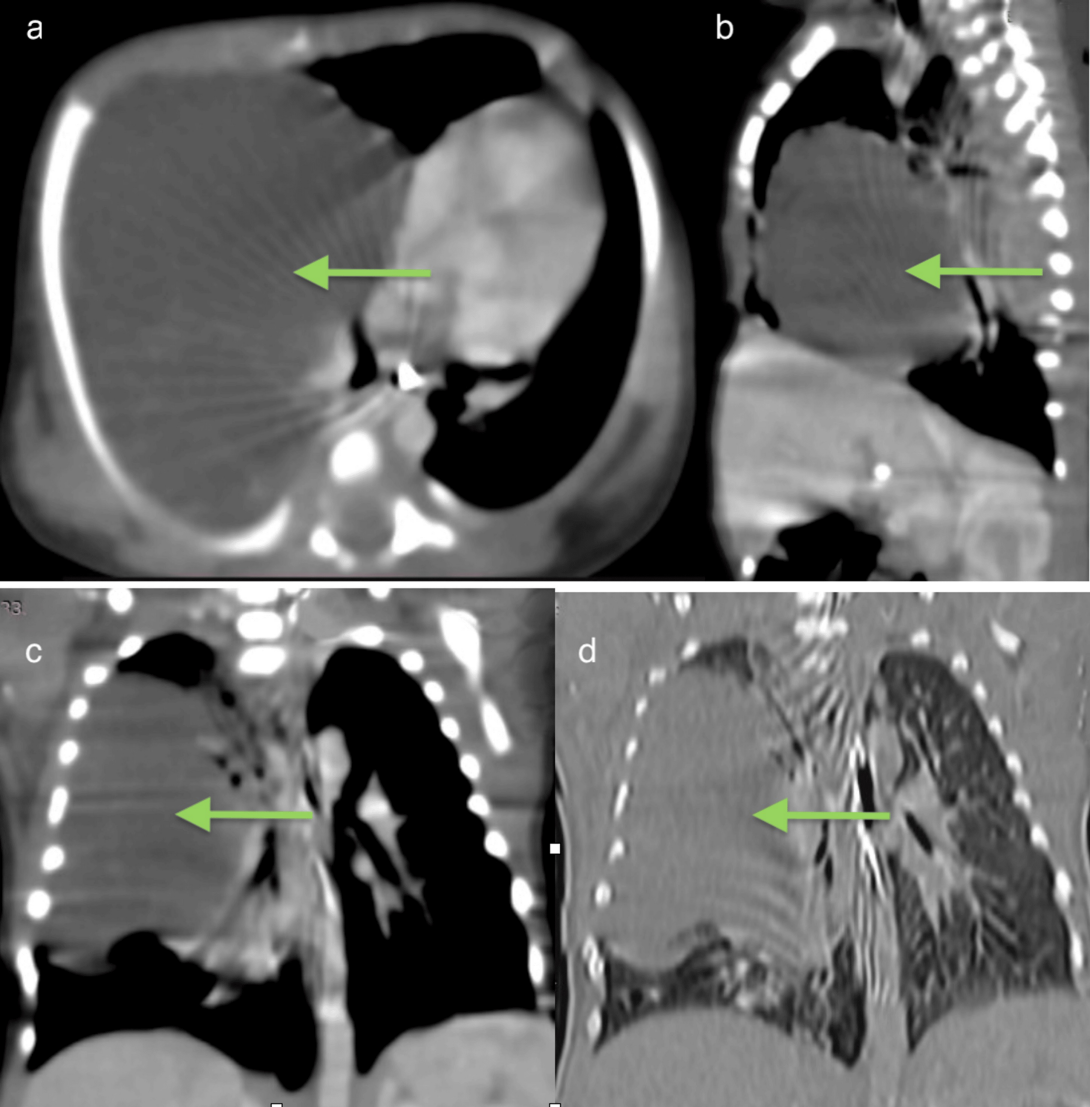
Multiplanar foetal contrast-enhanced CT images (axial, sagittal, and coronal views). (a) Axial image demonstrates a well-defined, large, multiloculated cystic lesion (arrow) involving the right middle lobe, causing splaying of the right upper and lower lobe bronchi with associated tracheomediastinal shift toward the left. Adjacent lung parenchyma shows collapse-consolidative changes. (b) Sagittal image demonstrates the craniocaudal extent of a multiloculated cystic lesion (arrow) involving the right middle lobe, with collapse-consolidation of adjacent lung. (c, d) Coronal reformatted images show a large multiloculated cystic lesion (arrow) in the right middle lobe, with separation of the right upper and lower lobe bronchi and resultant leftward tracheomediastinal displacement. Surrounding lung parenchyma demonstrates compressive collapse and consolidation.

**Figure 6:**
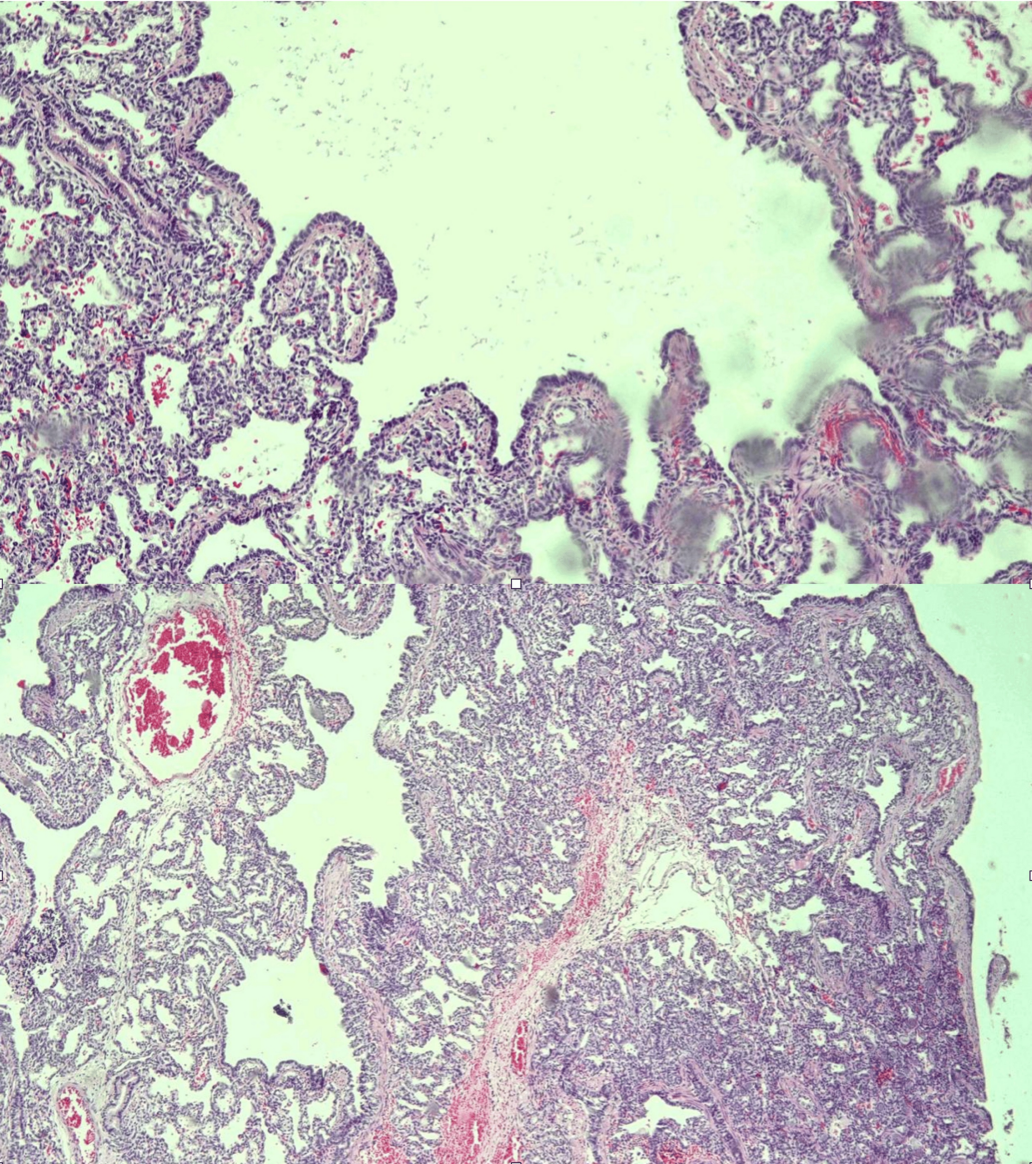
Histopathological examination of the lung section revealing a cystic lesion lined by ciliated columnar epithelium. The subepithelial tissue shows a few cystically dilated alveoli-like spaces lined by cuboidal cells.

Postnatal X-ray (Figure 4) and CT scan (Figure 5) were done, and these confirmed the diagnosis of CPAM. As respiratory distress persisted, the infant underwent right middle lobectomy on postnatal day nine. Histopathological examination of the lung section (Figure 6) revealed a cystic lesion lined by ciliated columnar epithelium. The subepithelial tissue showed a few cystically dilated alveoli-like spaces lined by cuboidal cells. No mucinous cell clusters were identified. The histological features were consistent with CPAM. Postoperatively, invasive mechanical ventilation was required, and the infant was extubated on the third postoperative day. The infant was subsequently supported with BCPAP for seven days and then weaned off onto low-flow nasal oxygen support.

## Discussion

CPAM is part of a heterogeneous group of congenital bronchopulmonary anomalies that also includes pulmonary sequestration, bronchial atresia, bronchogenic cysts, lobar agenesis, and polyalveolar lobe [1]. Pathologically, CPAM represents a developmental overgrowth of abnormal airway structures with variable cyst formation and absence of cartilaginous support within the cyst walls [3].

Although the precise aetiopathogenesis remains uncertain, altered epithelial-mesenchymal signalling during early foetal lung development is believed to contribute to lesion formation [5]. Reports of molecular abnormalities overlapping with paediatric lung tumours have raised concern regarding the potential for malignant transformation in long-standing lesions [6]. Prenatal ultrasound facilitates early detection. The CVR is obtained by dividing the CPAM volume by the head circumference. A CVR higher than 1.6 predicts risk of hydrops and neonatal complications, while a CVR lower than 0.9 suggests a favourable prognosis. Lesions demonstrating elevated CVR, macrocystic architecture, or mediastinal displacement are associated with increased risk of postnatal symptoms [7]. Progressive enlargement may result in hydrops fetalis secondary to compromised venous return and cardiac compression [4].

The imaging differential diagnosis of cystic lung lesions includes bronchogenic cysts, pulmonary sequestration (identified by systemic arterial supply), congenital diaphragmatic hernia, congenital lobar over-inflation, and localised cystic bronchiectasis [8]. Postnatal CT is essential for anatomical characterisation and surgical planning, particularly for identifying systemic arterial supply and differentiating CPAM subtypes. Solid-appearing lesions on CT may correspond to type III CPAM [8]. Foetal cystic lung lesions must also be distinguished from pulmonary lymphangiectasia and other congenital malformations [9].

Clinical presentation after birth ranges from respiratory distress and recurrent infections to incidental detection in asymptomatic individuals [4]. In selected high-risk cases, antenatal corticosteroid therapy has been shown to reduce lesion size and may eliminate the need for foetal surgical intervention [10]. The recommended regimen is two doses of betamethasone 12 mg administered intramuscularly before 32 weeks of gestation in cases with predominantly microcystic lesions or with CVR greater than 1.6 [10]. Pleuropulmonary blastoma and bronchioloalveolar carcinoma have been reported in association with CPAM, with type IV lesions showing clinicopathologic overlap with type I pleuropulmonary blastoma [6]. Early elective surgical resection at one to six months of age is often advocated to prevent infectious complications and mitigate malignancy risk [9].

## Conclusions

CPAM is a rare congenital lung malformation with a wide spectrum of antenatal and postnatal outcomes. Even in cases with high CVR, close monitoring for the development of hydrops, timely administration of antenatal corticosteroids, and early referral to specialised tertiary care for planned delivery can contribute to favourable outcomes, despite the need for postnatal surgical management.
